# Phytophagous insect oviposition shifts in response to probability of flower abortion owing to the presence of basal fruits

**DOI:** 10.1002/ece3.3426

**Published:** 2017-09-18

**Authors:** Shivani Jadeja, Brigitte Tenhumberg

**Affiliations:** ^1^ School of Biological Sciences University of Nebraska Lincoln NE USA

**Keywords:** context‐dependent strategy, flower abortion, host plant, oviposition behavior, phytophagous insect, *Tegeticula yuccasella*, *Yucca glauca*

## Abstract

Phytophagous insects use a wide range of indicators or associated cues to avoid laying eggs in sites where offspring survival is low. For insects that lay eggs in flowers, these unsuitable sites may be created by the host plant's resource allocation to flowers. In the sequentially flowering host plant, *Yucca glauca*, late‐opening distal flowers are more likely to be aborted in the presence of already‐initiated basal fruits because they are strong resource sinks. If flowers are aborted, all eggs of the phytophagous insect, *Tegeticula yuccasella*, within the flower die. We used the phytophagous insect *T. yuccasella* that lays eggs in and pollinates host plant *Y. glauca* flowers to test the hypothesis that phytophagous insect females are less likely to invest eggs in host plant flowers if basal fruits are present because they are more likely to be aborted. We also investigated potential predictors of arrival of *T. yuccasella* at inflorescences at the onset of flowering. These factors may influence a phytophagous insect's decisions to select oviposition sites. We carried out a behavioral experiment using wild‐caught *T. yuccasella* females on manipulated inflorescences with distal flowers with basal fruits and without fruits. As potential predictors of *T. yuccasella* arriving at inflorescences, we used floral display size and day of onset of flowering. In support of our hypothesis, our experimental results showed that *T. yuccasella* was significantly less likely to oviposit in distal flowers on inflorescences with basal fruits. We also found that *T. yuccasella* arrival was higher at inflorescences with larger floral display size and earlier in the flowering season. These findings uncover a novel indicator of unsuitable oviposition sites—the presence of basal fruits, that phytophagous insects use to make oviposition decisions. Further, our study contributes to the growing body of evidence that shows that females prefer sites that increase the probability of survival of their offspring.

## 1 INTRODUCTION

A wide range of phytophagous insects avoid laying eggs in host plants or plant parts that are unsuitable oviposition sites because they lead to a lower probability of offspring survival (Gripenberg et al., 2010; Mayhew, 1997; Renwick & Chew, 1994). To identify unsuitable oviposition sites, phytophagous insects use a variety of indicators, or tactile or chemical cues associated with those indicators. For example, for *Euura lasiolepis*, a shoot‐galling sawfly, offspring survival is lower in shorter shoots of the willow, *Salix lasiolepis,* that are more likely to fall off which kills the fly's offspring (Craig, Itami, & Price, 1989). Flies used shoot length as an indicator of suitability of oviposition sites and avoided shorter shoots (Craig et al., 1989). Some other indicators of unsuitable oviposition sites for phytophagous insects include the presence of specific plant secondary chemical compounds (Wennström et al., 2010), the presence of host‐marking pheromones laid during oviposition by conspecifics (Huth & Pellmyr, 1999), fungal infection on oviposition sites that increases the likelihood of abortion of oviposition sites (Biere & Honders, 2006), and age of plant parts where older plant parts may deteriorate before offspring can finish development (Heard, 1995).

For phytophagous insects that lay eggs in flowers, offspring survival is likely to be strongly dependent on how plants allocate resources to flowers. Plants abort flowers due to resource limitation and in many cases show a predictable pattern of flower abortion (Stephenson, 1981). For instance, in the sequentially flowering plant *Yucca glauca* (soapweed yucca), late‐opening distal flowers have a higher probability of abortion (Jadeja and Tenhumberg, unpublished data) possibly because early developing fruits are strong resource sinks. Further, the probability of flower abortion in *Y. glauca* increases with increasing number of basal fruits (Jadeja and Tenhumberg, unpublished data). For the phytophagous insects *Tegeticula* spp. (yucca moths) that lay eggs in and pollinate *Yucca* spp. flowers, all eggs within aborted flowers die. Abortion of flowers and young fruits causes mortality of 95.5% of the *Tegeticula* sp. eggs (Shapiro & Addicott, 2004). *Tegeticula* spp. are likely under selection to evolve and maintain oviposition strategies to reduce the loss of their eggs due to flower abortion (Wilson & Addicott, 1998). In the first part of this study, we explored the hypothesis that *T. yuccasella* uses the presence of basal fruits as an indicator of unsuitable oviposition sites. We made three predictions to test our hypothesis.

**Figure 1:**
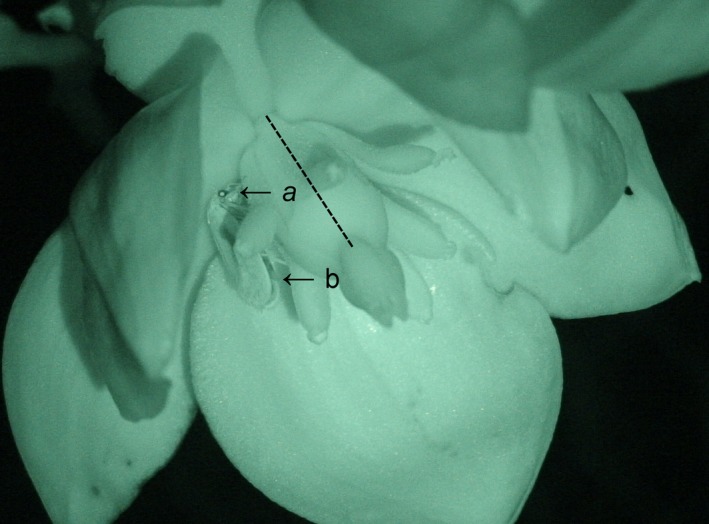
Wild‐caught *Tegeticula yuccasella* female (yucca moth) with a pollen ball under her head (arrow a), resting in a *Yucca glauca* flower during a behavioral trial. The moth is on the left‐hand side of the flower's ovary (dashed line running along its length). The posterior end of the abdomen of the moth (arrow b) bears an ovipositor that the moth inserts in the flower's ovary to lay an egg. This image was captured using the infrared light‐based night‐vision feature of a Sony^®^ Handycam video recorder in the HDR‐SR series

First, we predicted that, in the presence of basal fruits, *T. yuccasella* will be less likely to oviposit in distal flowers. Second, we predicted that, if *T. yuccasella* choose to oviposit in distal flowers with basal fruits present, the number of ovipositions will be fewer than in flowers without basal fruits. To test these predictions, we carried out a field behavioral experiment using wild‐caught *T. yuccasella* females (Fig. 1). Third, we predicted that the number of ovipositions will decrease with an increasing number of basal fruits. To test this prediction, we carried out an observational study using the number of *T. yuccasella* larvae emerging from naturally pollinated *Y. glauca* fruits as a proxy for the number of *T. yuccasella* ovipositions in flowers. In congeneric *T. altiplanella*, the number of ovipositions in flowers is positively correlated with the number of larvae emerging from fruits (Shapiro & Addicott, 2003).

Before phytophagous insect females decide to oviposit in a flower, they need to decide which inflorescences to explore as potential oviposition sites. Those decisions may be influenced by plant traits, environment, and how synchronized insect and plant phenology is. Hence, in the second part of this study, we explored factors predicting the arrival of *T. yuccasella* at inflorescences. Nectar‐feeding pollinators visit plants with larger floral displays more frequently than plants with smaller displays (Eckhart, 1991). In *Corydalis ambigua*, larger floral displays received both more frequent and longer visits by pollinators because larger floral displays likely signal higher rewards for pollinators (Ohara & Higashi, 1994). For *T. yuccasella,* larger floral displays likely indicate larger number of oviposition sites. Phytophagous insects may selectively visit inflorescences at certain locations within a population due to underlying microclimatic variables (Herrera, 1995; Thompson, 2001), such as shading or differences in temperature. In addition, arrival at inflorescences also depends on the synchrony between the phenologies of host plants and phytophagous insects. As *T. yuccasella* are difficult to observe away from inflorescences, we used the relationship between time of onset of flowering and arrival of *T. yuccasella* at inflorescences to gain insights into the synchrony between host plants and phytophagous insects. We carried out an observational study to explore the effect of three variables: floral display size, shading, and timing of onset of flowering on the probability of arrival of and number of *T. yuccasella* at *Y. glauca* inflorescences at onset of flowering.

## 2 MATERIALS AND METHODS

### 2.1 Study system

We used *Tegeticula yuccasella* (Family: Prodoxidae) and *Yucca glauca* (Family: Agavaceae), as our study system. Both species inhabit arid habitats across North and South America, and obligately depend on each other for their sexual reproduction. *Yucca* spp. produce racemose usually unbranched inflorescences consisting of 17 to 140 buds (Kingsolver, 1986; Svensson, Pellmyr, & Raguso, 2011; S. Jadeja, personal observation). The *Yucca* spp. flowering period is usually 15–30 days long (Powell, 1992) during which each flowering inflorescence opens subsets of flowers sequentially from the bottom‐up. Flowers are receptive for approximately 2 days upon opening. After pollination, *Tegeticula* spp. females lay their eggs in the flower's ovary, and, the hatching larvae feed on the host plant seeds (Riley, 1892). *Yucca* spp. populations retain on average less than 15% of their flowers as fruits (Addicott, 1998; Kingsolver, 1984; Pellmyr et al., 1997). Ninety‐five percent of the flowers that the plant aborts are aborted within a week after they open (Pellmyr & Huth, 1994). Causes of flower abortion include ovule damage by yucca moths during the process of oviposition (Marr & Pellmyr, 2003) and herbivory by florivorous beetles and their larvae (*Carpophilus sp*.) (Huth & Pellmyr, 1997, S. Jadeja, personal observation).

*Tegeticula yuccasella* enclose and emerge from the soil when their host plant is in flower. Adult females live for three to five days while males live for two to three days (Rau, 1945), with laboratory‐reared adults from our study site generally surviving about a week in the laboratory (S. Jadeja, personal observation). Upon emergence, moths seek host plant flowers, with the help of the flower's fragrance (Rau, 1945; Svensson et al., 2011). Moths are mostly active at night and rest inside the flowers during the day (Rau, 1945). *Tegeticula* spp. mate in *Yucca* spp. flowers. After mating, females engage in pollen collection, oviposition, and pollination behaviors. As female moths have a relatively short lifespan, we assume that they engage in oviposition and pollination behaviors soon after mating.

Female yucca moths seek suitable yucca flowers for oviposition. They prefer one‐ to two‐day‐old flowers (Riley, 1892). Females insert their ovipositor into the ovary and lay one egg during each insertion (Huth & Pellmyr, 1999; Pellmyr & Huth, 1994; Rau, 1945; Riley, 1892) and deposit host‐marking pheromones (Huth & Pellmyr, 1999; Kingsolver, 1984). An average of 37.5%–45% oviposition attempts fail (Huth & Pellmyr, 1999; Pellmyr & Huth, 1994; Segraves, 2003) possibly because of disturbance by other moths and insects, bad weather, or females may not properly insert their ovipositor into the ovary (Riley, 1892). After *Tegeticula* spp. females complete oviposition, they use their specialized mouthparts to push pollen down the opening in the stigma in multiple short up‐down motions. A female may oviposit multiple times in a flower, but each oviposition may not be followed by a pollination event (Addicott & Tyre, 1995; Tyre & Addicott, 1993). However, each pollination event is always preceded by at least one oviposition event (Addicott & Tyre, 1995). Females generally visit neighboring flowers and spend a longer time pollinating and ovipositing than moving between flowers on an inflorescence, suggesting that they minimize the distance between oviposition sites (Kingsolver, 1984; Pellmyr et al., 1997).

Within 7–10 days after oviposition, *Tegeticula* spp. larvae hatch and feed on the developing seeds within the maturing *Yucca* spp. ovary (Huth & Pellmyr, 1999). Surviving *Tegeticula* spp. larvae emerge from fruits 30 to 40 days after oviposition (Humphries & Addicott, 2004; Huth & Pellmyr, 1999). The emerging larvae burrow into the soil, form a cocoon, and remain dormant for at least one fall and winter (Riley, 1892). However, a large proportion of the larvae diapause for more than one year and for as long as four years (Riley, 1892).

### 2.2 Oviposition in response to the presence of basal fruits

#### 2.2.1 Obtaining inflorescence treatments

We manipulated *Y. glauca* inflorescences for use in behavioral trials to test whether *T. yuccasella* are less likely to oviposit in late‐opening distal flowers in the presence of basal fruits because they have a high likelihood of being aborted. We manipulated inflorescences following Jadeja and Tenhumberg (unpublished data) to obtain two inflorescence treatments—(1) inflorescences with late‐opening distal flowers and no basal fruits, and (2) inflorescence with late‐opening distal flowers and one to three basal fruits (see Appendix S1 for detailed methods). We protected 136 *Y. glauca* inflorescences that were yet to begin flowering from early May to mid‐June 2016 at a mixed‐grass prairie at the Cedar Point Biological Station (CPBS), Keith County, Nebraska, USA. We established inflorescences with one to three basal fruits by hand‐pollinating three to six bottom flowers of the inflorescence. Overall, we could use 23 of the 136 initially protected inflorescences in behavioral trials.

#### 2.2.2 Obtaining yucca moths

We used field‐collected *T. yuccasella* females for the behavioral trials. Wild‐caught moths may vary in their oviposition due to differences in age and experience, but this is unlikely to bias the results of the experiment because moths were randomly assigned to both inflorescence treatments. One of the advantages of using wild‐caught moths is avoiding possible artifacts introduced by rearing moths in the laboratory that may not be acclimatized to field conditions. We identified females by the dark brown scale‐less abdominal tip that is visible on the underside of the posterior end of their abdomen where the ovipositor is located. We collected moths by baiting them using cut *Y. glauca* inflorescences that we kept outdoors at the field station in buckets with water and plant food solution (Miracle‐Gro^®^) to keep the inflorescences fresh for longer. We replaced old inflorescences with newer cut inflorescences throughout the study period, as needed. Each evening we checked flowers on the cut inflorescences to collect *T. yuccasella* females.

We collected *T. yuccasella* females in 44 ml vials with holes in their caps for exchange of air. Upon collection, we visually checked the underside of the moths’ heads for the presence of a pollen ball. If a *T. yuccasella* female did not have a pollen ball, we allowed her to collect pollen in a smoothie cup with one to two fresh *Y. glauca* flowers. We checked the moths regularly until midnight to see whether they collected a pollen ball. We did not use moths without a pollen ball for the behavioral trials as they may not have mated or may show different oviposition strategies than moths with pollen balls. We preferred to use moths with pollen balls in behavioral trials on the same night they were collected. However, when that was difficult due to the availability of inflorescences of each treatment and time taken by previous trials (on average longer than 80 min per trial), we kept moths in the laboratory at the field station and used them in trials on subsequent nights. We housed the moths individually in 44 ml vials with a moist cotton roll to prevent dehydration. To maintain the moths’ circadian rhythm, we set the lighting in the laboratory to 12‐hr day light and 10‐hr night dark cycles, plus one hour each of gradual lighting and darkening during the mornings and evenings, respectively.

#### 2.2.3 Behavioral trials

We carried out behavioral trials during the mid to late *Y. glauca* flowering period between 6 and 20 June 2016. We conducted trials at night between 8:30 p.m. and 2:30 a.m. We aimed to run focal moths in trials of both treatments to account for individual‐level differences in oviposition. We alternated the order in which moths received both treatments to avoid confounding results with trial order. When possible, we conducted the second trial on the same night as the first trial with at least 20 min of rest period between the two trials. When a second trial was not possible on the same night due to unavailability of an inflorescence of the right treatment, lengthy previous trials, or stormy weather (affected sampling on two nights), we housed the moths in the laboratory as described earlier (see subsection “Obtaining yucca moths”), and used them during a subsequent sampling night, if possible. The time of the night when we run the trial (early versus late at night) may affect a moth's motivation and oviposition behavior. We avoided that from biasing our results by alternating the inflorescence treatment that was used at the beginning of a sampling night.

We carried focal *T. yuccasella* females with pollen balls in 44 ml vials to inflorescences in the field. After dark, we only used headlamps with dimmed red lights around collected moths as these are the least disturbing to the moths (Tyre & Addicott, 1993, S. Jadeja, personal observation). Prior to each trial, we lowered the mesh cage around the inflorescence with the desired treatment (with or without basal fruits), selected three topmost receptive and herbivory‐free experimental flowers, and removed the remaining flowers and buds. Then, we enclosed the inflorescence in a portable 101‐cm tall and 24‐cm diameter cylindrical trial cage made from a 0.18‐mm thick clear acetate sheet with fine mesh sleeves attached on both ends, and an opening with a mesh sleeve attached toward the bottom of the cage to introduce the focal moth. We tied the trial cage to the tomato cage surrounding the inflorescence, ensuring the sides of the trial cage did not touch the inflorescence stalk or the experimental flowers, and allowed ample space for the moth to move.

We introduced the focal moth in a vial from the opening toward the bottom of the trial cage and opened the vial which marked the start of a behavioral trial. We recorded the focal moth's activity using the infrared light‐based night‐vision video recording feature of Sony^®^ Handycam video recorders in the HDR‐SR series. In four trials, we made part of the observations visually or using a voice recorder due to technical difficulties in operating the video recorder. Later we scored the recordings for each trial and quantified the moths’ ovipositions. We considered the action of a focal moth inserting its ovipositor in the ovary of the flower and removing it after >30 s as one oviposition event because inserting the ovipositor for a shorter time would not have resulted in the deposition of an egg (Addicott & Tyre, 1995).

We discarded a trial if a focal moth did not exit the vial for 15 min from the start of the trial, and ended a trial if the moth did not begin ovipositing within 15 min from exiting the vial, did not oviposit 15 min after the last oviposition event or if a moth flew off the inflorescence stalk after its last oviposition event. We reused inflorescences where a focal moth did not oviposit in any of the flowers during a trial. Inflorescences where moths oviposited during a trial were not used in further trials to avoid host‐marking pheromones to influence the focal moth's oviposition behavior. Overall, we obtained first trials from 18 moths and second trials from 11 moths that exited their vials (see Table S1 for distribution of sample sizes).

### 2.3 Larval emergence in response to the presence of basal fruits

*Yucca glauca* open flowers sequentially from the bottom up. So the number of basal fruits is an index of the number of fruits already formed when the collected fruit was a flower. These fruits represent flowers that moths oviposited in and that the plants retained. We predicted that *T. yuccasella* decreases the number of ovipositions with increasing number of basal fruits on naturally pollinated inflorescences using larval emergence from a fruit as a proxy for the number of ovipositions in a flower. To check the suitability of our proxy, we constructed an Individual‐Based Model (Appendix S2). The model considered that *T. yuccasella* lays fewer eggs with increasing number of prior ovipositions (Huth & Pellmyr, 1999), *Yucca* spp. selectively abort flowers with a high number of *Tegeticula* spp. eggs (Humphries & Addicott, 2000; Pellmyr & Huth, 1994; Shapiro & Addicott, 2004). Flower abortion is unlikely affected by other sympatric *Tegeticula* sp. For instance, *T. corruptrix* occur later in the season and lay their eggs exclusively in fruits usually more than 2 weeks after pollination (Pellmyr, Leebens‐Mack, & Huth, 1996; S. Jadeja, personal observation), which is after the period when plants abort flowers and early fruits (Pellmyr & Huth, 1994; S. Jadeja, personal observation). Our simulation results show that only when moths decrease the number of ovipositions with increasing number of basal fruits can we expect a negative relationship between number of emerging larvae and number of basal fruits (Fig. S2.3).

To test whether *T. yuccasella* in the field vary the number of oviposition in response to the presence of basal fruits, we collected all the full‐grown fruits from the top third flowers of haphazardly selected naturally pollinated *Y. glauca* inflorescences in late June and July in the years 2014, 2015, and 2016. Those flowers opened mid to late in the flowering season and had similar display sizes (S. Jadeja, personal observation). The fruits came from a 55 × 25 m patch of *Y. glauca* on the North‐East slope of the Kingsley dam at Lake McConaughy, Keith County, Nebraska. This patch is 5 km from CPBS where we carried out the behavioral field experiment. We identified the top fruits using the relative position of the fruits and aborted flowers. When a flower aborts, its stalk (pedicel) is left behind and can be used to determine the flower's position on the inflorescence prior to abortion. We labeled each collected fruit, kept them in individual containers at room temperature over the fall, and recorded the number of emerging *Tegeticula* spp. larvae from each fruit. Next, we quantified the number of fruits basal to each top fruit.

Larval emergence from *Y. glauca* fruits is low and highly variable (S. Jadeja, personal observation), and fruiting from the top third flowers is not very common in natural populations, particularly when inflorescences have already matured basal fruits (Jadeja and Tenhumberg, unpublished data). Therefore, in 2016, we increased our sample size by collecting top fruits from 18 inflorescences from outside the patch, but from within the same area. These fruits came from both inflorescences with and without basal fruits.

At our study site, a nonpollinating congener of *T. yuccasella*—*T. corruptrix* lays eggs in fruits and has larvae that are morphologically indistinguishable from the pollinating *T. yuccasella* larvae. In contrast, the adults of these moth species can be easily morphologically distinguished. To determine the relative proportions of *T. yuccasella* and *T. corruptrix* larvae at our study site, we used reared larvae collected from *Y. glauca* fruits in summer 2014 and 2015 as part of a different study. We allowed larvae to burrow in soil‐filled cans. We covered the cans with cling wrap with holes to allow exchange of air but prevent excessive loss of soil moisture. We maintained the cans at room temperature (21–27°C) during the fall, spring, and summer, and colder temperatures (5°C or 18°C) during the winter, except during transportation when it was not feasible to regulate the temperature. We added a small quantity of water to the cans approximately once every two months to moisten the soil. Adults from some of the larvae collected in 2014 emerged in 2015 and 2016, and adults from some of the larvae collected in 2015, emerged in 2016. After moths enclosed in 2016, we terminated larval rearing.

### 2.4 Predictors of *T. yuccasella* arrival at onset of flowering

Each morning of the flowering season, we checked inflorescences protected for the field experiment and noted when the first flower on an inflorescence opened (onset of flowering). In addition, we recorded (1) how many *T. yuccasella* arrived at the inflorescence, (2) how many flowers opened as an index of size of the floral display, (3) the basal diameter of the rosette from which the inflorescence was emerging as an index of plant size, (4) the straight‐line distance to the nearest red cedar tree (*Juniperus virginiana*) that may provide an index of the presence of shade over the inflorescence, and (5) the Universal Transverse Mercator (UTM, Zone 14T, datum WGS 84) Easting and Northing coordinates to account for spatial autocorrelation, if any.

*Tegeticula yuccasella* rested on mesh sleeves of protected inflorescences during the day as the sleeves prevented them from accessing the flowers. We considered *T. yuccasella* on mesh sleeves as having arrived at the inflorescences. This was performed before we manipulated inflorescences for the field experiment. We obtained *T. yuccasella* arrival data from 111 of the 136 initially protected inflorescences after discarding 25 inflorescences that either dried or were damaged before onset of flowering. These 111 inflorescences were located over a distance of 352 m along the West‐East direction (UTM Easting, Zone 14T, datum WGS 84) and 844 m along the North‐South direction (UTM Northing, Zone 14T, datum WGS 84). The elevation ranged from 971 m to 1023 m above sea level.

### 2.5 Statistical analysis

#### 2.5.1 Oviposition in response to presence of basal fruits

We used a generalized linear mixed‐effects model (GLMM) with binomial error distribution to determine whether the probability of *T. yuccasella* oviposition in behavioral trials differed between treatments and trial order. The response variable was the proportion of flowers with at least one oviposition. Next, we used a linear mixed‐effects model (LMM) to determine whether the number of *T. yuccasella* oviposition in behavioral trials with at least one oviposition differed between treatments and trial order. The response variable was the log‐transformed number of ovipositions in a trial with at least one oviposition. The predictor variables were the presence of basal fruits (inflorescence treatment) and trial order (first or second trial), and the random effects were moth identity and trial night. We used backward model selection to identify the minimum adequate model for our experimental data using a significance cutoff of 0.05 (see Tables S2 and S3 for the results from the full models).

#### 2.5.2 Larval emergence in response to the presence of basal fruits

We analyzed the number of larvae emerging from fruits from top third flowers using GLMMs with a Poisson error distribution with inflorescence identity as a random effect. The fixed effects were number of basal fruits and year. Year was treated as a categorical variable.

#### 2.5.3 Predictors of *T. yuccasella* arrival at onset of flowering

We used Spearman's rank correlation analysis to quantify correlations between all factors we measured. In our statistical models, we only included predictor variables that were not highly correlated (maximum correlation coefficient was less than 0.5 for predictor variables in each model). Further, we checked for spatial autocorrelation in the probability and number of moths arriving at inflorescences and found no significant spatial autocorrelation. There was no significant spatial autocorrelation in the number (Moran's *I* = 0.018, *p* = .4) and probability (Moran's *I* = 0.18, *p* = .2) of moths arriving at inflorescences at onset of flowering (see Fig. S3.1a,b for semivariograms). Hence, we did not consider the coordinates of the inflorescences in our analysis.

We analyzed the probability of moths arriving at onset of flowering using a generalized linear model (GLM) with binomial distribution of errors. The response variable for the full model was the presence/absence of moths at onset of flowering and predictor variables were number of open flowers, day of onset of flowering, basal diameter, and distance to nearest tree (see Tables S7 and S8 for model selection details). We analyzed the number of moths arriving at inflorescences conditional on moths being present using a generalized additive model (GAM) with Poisson's distribution of errors to capture the complex nonlinear response of the number of moths arriving and day of onset of flowering. The response variable for the full model was the number of moths at the inflorescence, and the predictor variables were number of flowers open, smooth splined day of onset of flowering, basal diameter, and distance to nearest tree (see Tables S10 and S11 for model selection details). In both models, we considered the date the first inflorescence started flowering as the first day of onset of flowering.

For the observational data, we used an information theoretic approach (Burnham & Anderson, 2002) to identify the final model for the probability of arrival and number of moths arriving at onset of flowering. To account for the small sample sizes, we used the corrected Akaike Information Criterion (AICc). We show the effect of each predictor variable on the response variable in the final model by holding other predictor variables at their median values.

We carried out all statistical analyses in R version 3.3.2 (2016‐10‐31) (R Core Team 2016), using packages lme4 (Bates et al., 2015), mgcv (Wood, 2016), and nlme (Pinheiro et al., 2016).

## 3 RESULTS

### 3.1 Oviposition in response to presence of basal fruits

*Tegeticula yuccasella* oviposited at least once in 55% of the trials (*n* = 29 trials). Of these, 63% of the trials were on inflorescences without basal fruits. The total number of *T. yuccasella* ovipositions in trials with at least one oviposition ranged from 3 to 109 ovipositions across the three experimental flowers, with an average of 19 ± 7 (mean ± *SE*) ovipositions (*n* = 16 trials). The number of ovipositions in individual flowers with at least one oviposition during the experiment ranged from 2 to 52 ovipositions with an average of 12 ± 2 (mean ± *SE*) ovipositions (*n* = 25 flowers across 16 trials).

**Figure 2:**
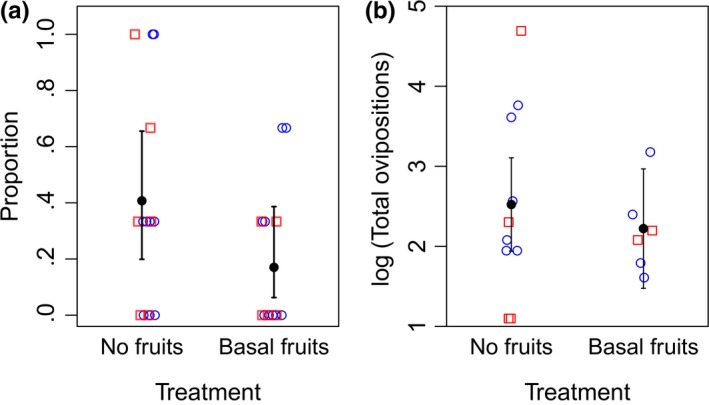
(a) The proportion of flowers with at least one oviposition is significantly lower on inflorescences with the presence of one to three basal fruits than on inflorescences without basal fruits. (b) There is no significant difference in the total number of ovipositions in trials with at least one oviposition between inflorescences with basal fruits and inflorescences without basal fruits. Points are jittered along their *x*‐axis to visualize overlapping points. Open circles are first trials and open squares are second trials. Filled points and error bars are model predicted means and 95% CIs, respectively, from the simplified models with only the presence of basal fruits as a predictor variable (*n* = 29 trials)

Our analysis showed that the presence of basal fruits significantly reduced the proportion of flowers with at least one oviposition. Moths oviposited on average in 1–2 of 3 flowers when no fruits were present and in 0–1 of 3 flowers when basal fruits were present (*p* = .048, Fig. 2a, Table S4). Additionally, the presence of basal fruits did not significantly reduce the total number of ovipositions in trials with at least one oviposition (*p* = .61, Fig. 2b, Table S5).

### 3.2 Larval emergence in response to the presence of basal fruits

**Figure 3:**
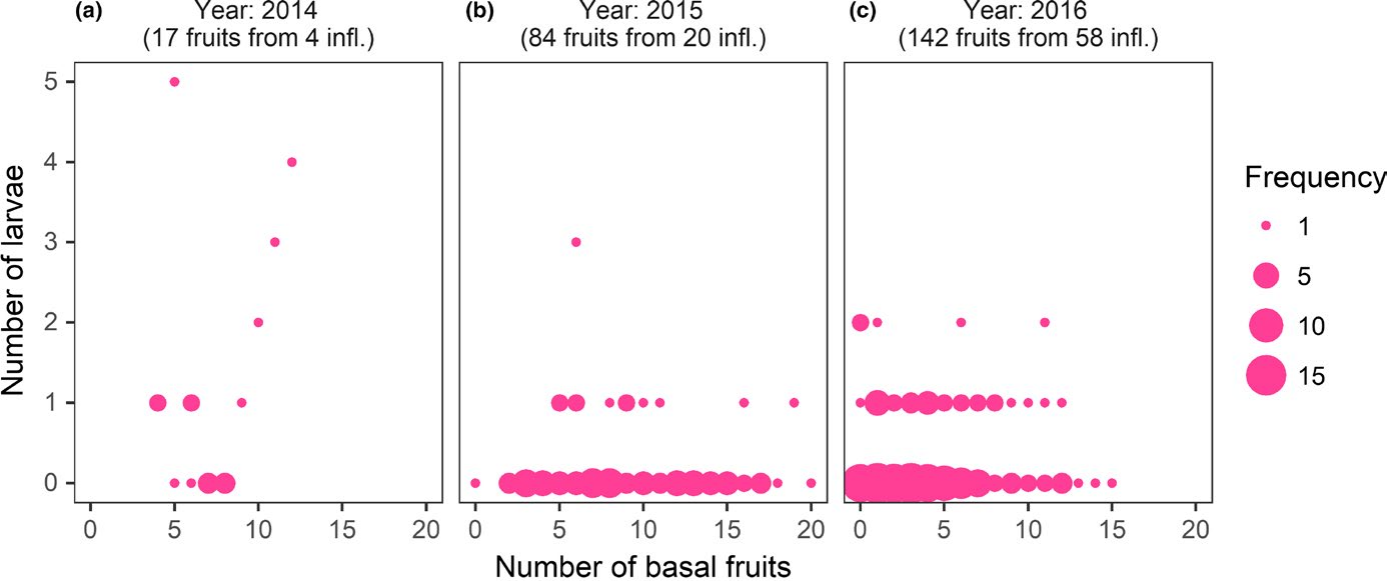
The number of larvae emerging from fruits from top third flowers is not predicted by the number of basal fruits across 3 years (a,b,c). Points are fruits, and the size of the points is proportional to the frequency of observations. Sample sizes are shown in parentheses above figure panels where infl. stands for inflorescences

Overall, not many larvae emerged from fruits. In only 22% of the top fruits (*n* = 243 fruits), one or more larvae developed successfully. The average number of larvae emerging from fruits of top third flowers was 0.3 ± 0.04 (mean ± *SE*,* n* = 243 fruits). In all three years, the number of basal fruits did not affect the number of larvae emerging from top fruits (*p* > .7, Fig. 3a–c, Table S6). Adult moths emerging from reared larvae showed that the proportion of nonpollinating moths (*T. corruptrix*) was 11% (*n* = 28 moths) and 4% (*n* = 24 moths) in 2015 and 2016, respectively.

### 3.3 Predictors of *T. yuccasella* arrival at onset of flowering

Inflorescences opened 7.5 ± 0.5 (mean ± *SE*) flowers with a maximum of 25 flowers at onset of flowering (*n* = 111 inflorescences). The first inflorescence started flowering on 26 May 2016 and the last inflorescence started flowering on 16 June 2016. Each inflorescence flowered for about 1 to 2 weeks.

**Figure 4:**
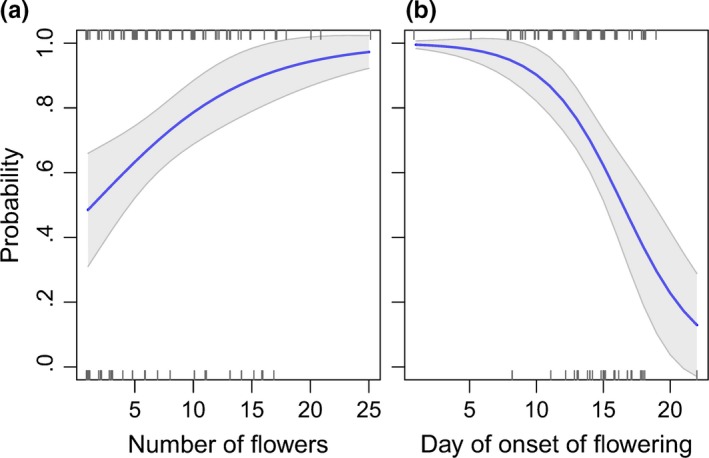
The probability of moths arriving at inflorescences at onset of flowering was (a) positively correlated with the number of flowers open at onset of flowering, and (b) negatively correlated with the day of onset of flowering. In 2016, the first day of onset of flowering (day 1) was May 26. Lines and shaded areas show model predicted means (solid lines) and 95% CIs, when the other variables are at their median value. Rugs show observed the presence and absence of moths (*n* = 111 inflorescences)

The probability of *T. yuccasella* arriving at onset of flowering increased significantly with increasing number of flowers open, and decreased over the flowering season (Fig. 4a,b, Table S9). There was a .48 probability of moths arriving at inflorescences with one open flower, which almost doubled to .97 when 25 flowers were open (*p* = .003, Fig. 4a). Further, there was a greater than .90 probability of moths arriving at inflorescences with onset of flowering within the first 10 days of the flowering season. However, the probability of arrival reduced to less than .2 at the end of the flowering season (*p* < .0002, Fig. 4b).

**Figure 5:**
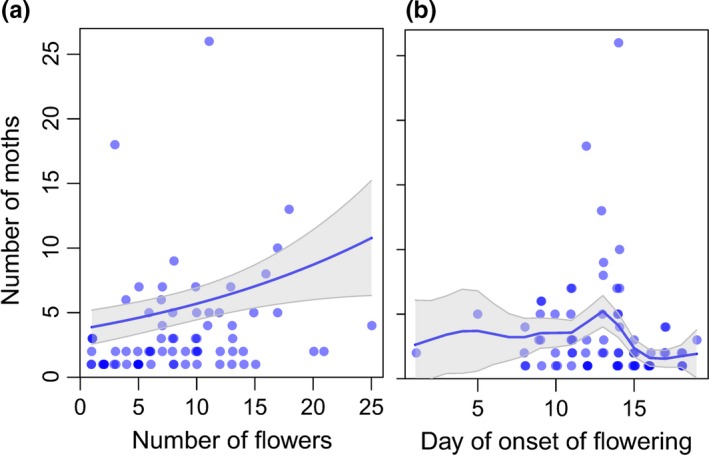
On inflorescences where moths arrived at onset of flowering, the number of moths arriving (a) increased with increasing number of flowers open at onset of flowering, and (b) changed in a complex nonlinear pattern with day of onset of flowering. In 2016, the first day of onset of flowering (day 1) was May 26. Points are inflorescences. Darker points are overlapping points. Lines and shaded areas show model predicted means (solid lines) and 95% CIs when the other variable is held at its median value (*n* = 76 inflorescences)

On inflorescences visited by *T. yuccasella*, the average number of moths arriving was 3.5 ± 0.5 (mean ± *SE*) moths with a maximum of 26 moths. The number of *T. yuccasella* arriving significantly increased with increasing number of open flowers, and significantly changed nonlinearly over the flowering season (Fig. 5a,b, Table S12). The number of moths arriving was 4 on inflorescences with one open flower, and more than tripled to 11 on inflorescences with 25 open flowers (*p* = .001, Fig. 5a). Further, the number of moths arriving peaked close to the middle of the flowering season on the 13th day with 5 moths arriving on average. The number of moths more than halved to less than 2 moths arriving at inflorescences with the most delayed onset of flowering (*p* < .0001, Fig. 5b).

## 4 DISCUSSION

### 4.1 Oviposition in response to the presence of basal fruits

*Yucca glauca* flowers are more likely to be aborted in the presence of basal fruits (Jadeja and Tenhumberg, unpublished data). Further, all *Tegeticula* spp. eggs in flowers that are later aborted die (Huth & Pellmyr, 1999; Shapiro & Addicott, 2004). Hence, we hypothesized that *T. yuccasella* will be less likely to invest eggs in distal flowers on inflorescences with basal fruits. As expected, the probability of *T. yuccasella* oviposition was lower in flowers on inflorescences with basal fruits. These results support our prediction that *T. yuccasella* will avoid laying eggs in flowers with a higher probability of abortion. Possible proximate cues for *T. yuccasella* to reject distal flowers with basal fruits as oviposition sites include tactile and/or chemical cues from fruits and/or flowers. There is overwhelming empirical evidence to show that many lepidopterans use multiple plant‐based cues to identify suitable oviposition sites and reject unsuitable ones, both within and between host plant species (reviewed in Renwick & Chew, 1994; Wennström et al., 2010; Ryuda et al., 2013; Mukae et al., 2016). Identifying specific cues that females use to respond to the presence of basal fruits is an avenue for further research.

A strategy to avoid oviposition in distal flowers may benefit *T. yuccasella* and similar phytophagous insect females in different ways. First, it may save females from losing a large proportion of their eggs in years with a large number of inflorescences with basal fruits. This benefit would be large during certain years and at certain sites in host plants like *Yucca* spp. where the frequency of distal flowers with and without basal fruits may vary across space and time because fruiting is highly resource limited (Humphries & Addicott, 2004; Huth & Pellmyr, 1997; Pellmyr & Huth, 1994) and variable (Addicott, 1998; Kingsolver, 1986). Second, short‐lived females like *Tegeticula* spp. that are time limited in their ability to deposit eggs may benefit from avoiding the opportunity costs of spending time ovipositing in flowers that are unlikely to form fruits. Likewise, females of an egg‐limited species in the same scenario would also benefit from selecting sites that are more likely to give each egg a higher chance of survival.

The number of ovipositions in flowers accepted as oviposition sites is another measure of the female's egg investment in flowers. We predicted that in our experiment, if *T. yuccasella* choose to lay eggs in flower with basal fruits, they will lay fewer eggs than in flowers without basal fruits. However, contrary to expectations, *T. yuccasella* did not lay significantly fewer eggs in flowers on inflorescences with basal fruits. It is possible that *T. yuccasella* do not decrease the number of eggs they lay in response to the presence of basal fruits. This suggests that their strategy is limited to determining whether a flower is a suitable oviposition site and does not involve determining number of eggs to oviposit. Alternatively, it is likely that we could not detect the expected pattern due to a high variation in the number of ovipositions among trials. The number of eggs laid may vary due to differences in the number of ovipositions by wild‐caught moths. For example, wild‐caught moths may have varied in their age‐related oviposition strategy. Older moths nearing the end of their life may oviposit more eggs in each flower they visit, which may increase variation in the ovipositions we observed. An example of the effect of life expectancy on oviposition behavior comes from parasitic wasps (Roitberg et al., 1992, 1993). Parasitic wasps have a low rate of ovipositing in already parasitized hosts. However, when parasitic wasps perceive they are near the end of their life, they increase their rate of ovipositing in already parasitized hosts.

Our study shows a novel way phytophagous insects can increase their fitness—a tendency to avoid ovipositing in distal flowers in the presence of basal fruits because they have a higher probability of abortion (Jadeja and Tenhumberg, unpublished data). *Tegeticula yuccasella* is also an obligate pollinator of *Y. glauca*. The consequences of such oviposition behavior on the complex eco‐evolutionary dynamics between mutualist partners are beyond the scope of this study. However, our results suggest that *T. yuccasella* has evolved an oviposition strategy that increases the number of surviving larvae which is in line with theory and empirical studies that show that phytophagous insect females prefer to oviposit in sites that are better for larval performance and survival (Gripenberg et al., 2010; Mayhew, 1997). Our investigation is also in line with egg‐laying site choice of female anurans that prefer to oviposit in ponds with a faunal composition that provides the best chances of survival for their offspring (Resetarits, 1996).

Ideally, we would have designed an experiment allowing females to choose between flowers with and without basal fruits in a trial to identify a female's oviposition preference. However, this was not possible with inflorescences attached to plants in the field because the inflorescences were very often located many meters away from each other. In order to present a female moth with both inflorescence treatments simultaneously in a choice experiment, we would have had to cut inflorescences and place them besides each other in a trial cage. Cutting inflorescences could have affected the chemical cues used by the female moth to assess a flower's probability of abortion. To avoid the risk of losing chemical cues of the flower's probability of abortion, we used inflorescences attached to the plants in the field that prevented us from designing a choice experiment.

### 4.2 Larval emergence in response to the presence of basal fruits

The number of larvae emerging from fruits is an index of the number of *Tegeticula* sp. ovipositions (Shapiro & Addicott, 2003). As the probability of flower abortion decreases with increasing number of basal fruits (Jadeja and Tenhumberg, unpublished data), we originally expected fewer larvae to emerge from distal fruits with increasing number of basal fruits. However, in our field experiment, we did not detect a significant decrease in the number of oviposition in the presence of basal fruits. In line with our experimental results, our field observational study shows that the number of larvae emerging from fruits of naturally pollinated top third flowers did not decrease with increasing number of basal fruits.

There are three possible explanations for the absence of a relationship between the number of emerging larvae and number of basal fruits. First, the probability of an egg to survive in a flower may influence a *T. yuccasella* female's decision to accept a flower as an oviposition site, but once a flower has been accepted, the female may not decrease the number of ovipositions in response to increasing number of basal fruits. Hence, when flowers with basal fruits are retained, we do not see a decrease in the number of larvae emerging from their fruits. Second, *T. yuccasella* larvae may experience higher density‐dependent larval mortality in fruits without basal fruits where we expected a larger number of larvae. This may result in the same number of larvae independent of the number of ovipositions. A study has documented density‐dependent larval mortality in congeneric *T. altiplanella* (Shapiro & Addicott, 2003).

Third, the true pattern of larval emergence may be masked by our inability to morphologically distinguish larvae of pollinating *T. yuccasella* and congeneric nonpollinating *T. corruptrix*. It is possible that later‐occurring, nonpollinating *T. corruptrix* lay more eggs in fruits with basal fruits, or their larvae have a higher probability of survival in fruits with basal fruits due to weak competition with *T. yuccasella* larvae. This would result in a negative relationship between the number of *T. yuccasella* and *T. corruptrix* larvae emerging from fruits. As a result, there may be no overall differences in the total number of larvae emerging as number of *T. yuccasella* larvae increase. For instance, in two of three years, the number of pollinating and nonpollinating *Tegeticula* spp. larvae emerging from *Y. filamentosa* fruits was negatively correlated (Marr, Brock, & Pellmyr, 2001). However, the presence of the *T. corruptrix* larvae is unlikely to explain the results from our study because *T. corruptrix* larvae occurred in low frequency at our study site. Of the laboratory‐reared adult moths that eclosed in 2015 and 2016 only 11% (3 of 28 moths) and 4% (1 of 24 moths) were nonpollinating *T. corruptrix*. Therefore, we believe it is unlikely that our inability to morphologically distinguish larvae from pollinating and nonpollinating *Tegeticula* spp. has contributed to no relationship between the number of larvae emerging and number of basal fruits.

### 4.3 Predictors of *T. yuccasella* arrival at onset of flowering

Both the probability and number of moths arriving at inflorescences increased with larger floral display sizes at onset of flowering. Pollinator preference for plants with larger floral displays has been well‐established in nectar‐feeding pollinators (Buide, 2005; Eckhart, 1991; Ohara & Higashi, 1994; Thompson, 2001). We show this pattern holds true for the non‐nectar seeking *T. yuccasella* too. Larger floral displays with more open flowers may increase the probability of a moth finding an inflorescence through visual and/or chemical cues. The presence of a larger number of flowers may also increase the probability of finding receptive flowers that have not yet been oviposited in. In addition, larger floral displays may increase the likelihood of finding mates because it attracts a larger number of moths.

Finally, both the probability and number of *T. yuccasella* arriving were very low on inflorescences with late onset of flowering. This result may be explained by a mismatch between the availability and abundance of the *T. yuccasella* and flowering host plants. In our study, *T. yuccasella* abundance may have been low later in the flowering season. If so, we expect *T. yuccasella* arrival to be independent of day of onset of flowering in years with a greater synchrony between the availability of the *T. yuccasella* and host plants. Alternatively, this result may be explained by the presence of other inflorescences that started flowering earlier in the flowering season that co‐occur with inflorescences with late onset of flowering. Therefore, late in the flowering season, more competition among a larger number of flowering inflorescences may reduce the chances of finding moths at a particular inflorescence. Further, after onset of flowering inflorescences usually have larger floral displays than at onset of flowering (S. Jadeja, personal observation). We have already shown in this study that *T. yuccasella* are more likely to arrive at inflorescences with larger floral displays. Therefore, the low probability and number of *T. yuccasella* on inflorescences with late onset may also be due to *T. yuccasella* preferring larger floral displays of already flowering inflorescences.

In conclusion, the result from our observational study shows that floral display size and timing of onset of flowering are likely important in influencing *T. yuccasella* decisions to arrive at inflorescences. These factors may also influence the female's decisions to invest eggs in inflorescences and the distribution of eggs and fruiting success across inflorescences in a flowering season. It is likely that these results are applicable to other phytophagous insect species.

## DATA ACCESSIBILITY

Data and code for Individual‐Based Model, available from the Dryad Digital Repository: http://dx.doi.org/10.5061/dryad.1gk77.

## AUTHOR CONTRIBUTIONS

SJ and BT designed the study, developed the methodology, performed the analysis, and wrote the manuscript. SJ collected the data.

## CONFLICT OF INTEREST

The authors declare that they have no conflict of interests.
