# Supplementary material for: Phytophagous insect oviposition shifts in response to probability of flower abortion owing to the presence of basal fruits
Source: Ecol Evol. 2017 Sep 18;7(21):8770–9. doi: 10.1002/ece3.3426 (PMC5677471; doi:10.1002/ece3.3426)
Supplement: Supplementary file 1 [file ECE3-7-8770-s001.docx]

**SUPPLEMENTARY MATERIAL**

**Appendix S1:**

**Details of methods used to obtain inflorescence treatments**

We selected 136 *Y. glauca* inflorescences that were yet to begin flowering from early May to mid-June 2016 at a mixed-grass prairie at the Cedar Point Biological Station (CPBS), Keith County, Nebraska, USA. We protected the inflorescences from deer herbivory using tomato cages with sides wrapped with 2.54 cm hex netting. When the growing inflorescences were strong enough, we covered them with long cylindrical mesh cages made from fine tulle fabric sleeves with wire rings of approximately 20.5 cm diameter to prevent yucca moths from visiting flowers and florivorous beetles (*Carpophilus sp.*) from damaging flowers. Before placing the mesh cages, we removed all visible florivorous beetles from the inflorescence. At the onset of flowering, we alternately assigned each inflorescence to one of the two treatments.

We established inflorescences with one to three basal fruits by hand-pollinating three to six bottom flowers of the inflorescence. We used pollen from fresh donor flowers from protected inflorescences at least 25 m away from recipient plants. We collected donor flowers in a cooler with a small quantity of ice to keep the flowers cool, and utilized their pollen within three hours of collection. We transferred pollen from one anther lobe of a donor flower to the stigmatic opening of a recipient flower using a toothpick. Thereafter, we used a size 00 brush to push the pollen inside the stylar canal. All hand-pollinated flowers received pollen from the same pollen donor. We thoroughly cleaned both the toothpick and the brush between pollen donors to prevent transfer of pollen of mixed genotypes.

Thirty-five percent hand pollinations resulted in fruits. If hand-pollinated inflorescences did not retain any basal fruits, we considered it a treatment without basal fruits, and we reassigned the treatments on subsequently flowering inflorescences to ensure a sufficient number of inflorescences of each treatment were available at any time during the flowering season.

Inflorescences were checked daily to determine whether they were ready to be used in a behavioral trial. An inflorescence was considered ready for use in a behavioral trial when at least three undamaged receptive (1-2 days old) flowers were available from the top third buds of the inflorescence.

We discarded inflorescences with damage from deer and with florivorous beetles left inside mesh cages. Further, we also discarded inflorescences that dried before flowering or finished flowering sooner than we could use them in a behavioral trial. In 2016, flowering was about 10 days shorter than the previous year likely due to a warmer summer (Jadeja, personal observation). Overall, we could use 23 out of the 136 initially protected inflorescences in behavioral trials.

**Appendix S2:**

**Description of Individual-Based Model**

We developed an Individual-Based Model (IBM) to support our prediction that with increasing number of basal fruits fewer yucca moth larvae will emerge from distal fruits because of fewer ovipositions in those flowers with increasing number of basal fruits. We simulated flowering, moth arrival and oviposition, and fruiting in sequentially flowering inflorescences. Our model assumes that oviposition is a hierarchical process. After arriving at a flower, a female first decides if the flower is suitable for oviposition at all, and if it is then the female decides how many eggs to oviposit. This process is consistent with the bimodal distribution of number of ovipositions from our experiment described in the main text.

**Binomial probability of ovipositing at least one egg, *P_ovi_*.** Yucca moths are less likely oviposit in flowers with prior ovipositions (Huth & Pellmyr 1999) probably to reduce competition for resources and because flowers containing many eggs have a higher chance of being aborted (Pellmyr & Huth 1994; Humphries & Addicott 2000; Shapiro & Addicott 2004). Hence, we assumed that when a moth arrives at a flower, her probability of ovipositing at least one egg, *P_ovi_*, will decrease with increasing number of prior ovipositions, *E*. To implement this behavior we used the following sigmoidal function that we adapted from Louda et al. (2011) (Figure S2.1a):

$$P_{ovi}\left( E \right)= e^{-\left( \frac{E}{12} \right)^{3}} (equation 1)$$

In some simulations, we assumed that moth oviposition probability will decrease linearly with increasing number of basal fruits because previous work (Jadeja and Tenhumberg, submitted) indicated that flower abortion is higher in the presence of basal fruits, *B* (Figure S2.1b).

$$P_{ovi}\left( E,B \right)= e^{-\left( \frac{E}{12} \right)^{3}}\times\left( 1- \frac{B}{29} \right) (equation 2)$$

**Number of ovipositions, λ**: The model assumes that the number of ovipositions is Poisson distributed with mean $\lambda$ = 6. We explored two scenarios. One scenario assumes that the number of ovipositions is independent of the presences of basal fruits. The other scenario assumes that the number of ovipositions decreases with increasing number of basal fruits, *B*.

$$\lambda(B)=6 \times\left( 1-\frac{B}{29} \right) (equation 3)$$

In *Yucca glauca* less than 15% of the flowers are retained (Kingsolver 1984; Pellmyr et al. 1997; Addicott 1998). Our model assumes that the probability of retaining a flower decreases with the number of ovipositions, *E*, and with increasing number of basal fruits, *B* (equation 4, Figure S2.2).

$$P_{ret}\left( E,B \right)= e^{-\left( \frac{E}{20} \right)^{5}}\times\left( 1- \frac{B}{29} \right) (equation 4)$$

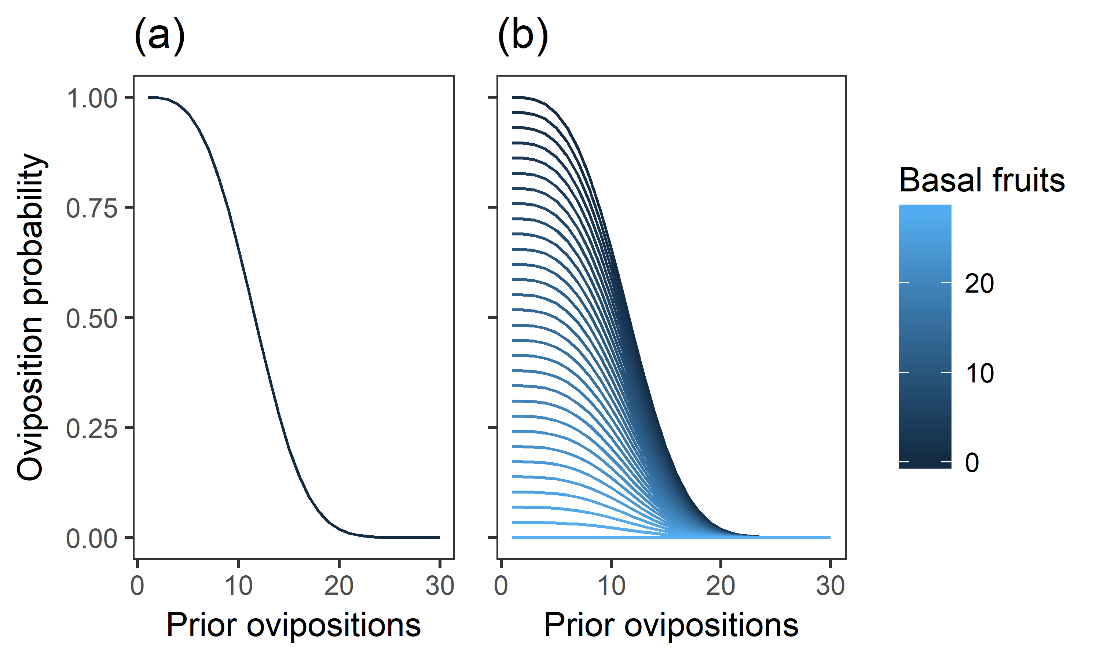


**Figure S2.1.** Probability of ovipositing at least one egg in flowers with increasing number of prior ovipositions from previous moth visits. In (a) oviposition probability only depends on number of prior ovipositions (equation 1), and in (b) oviposition probability decreases linearly with the number of basal fruits (equation 2).


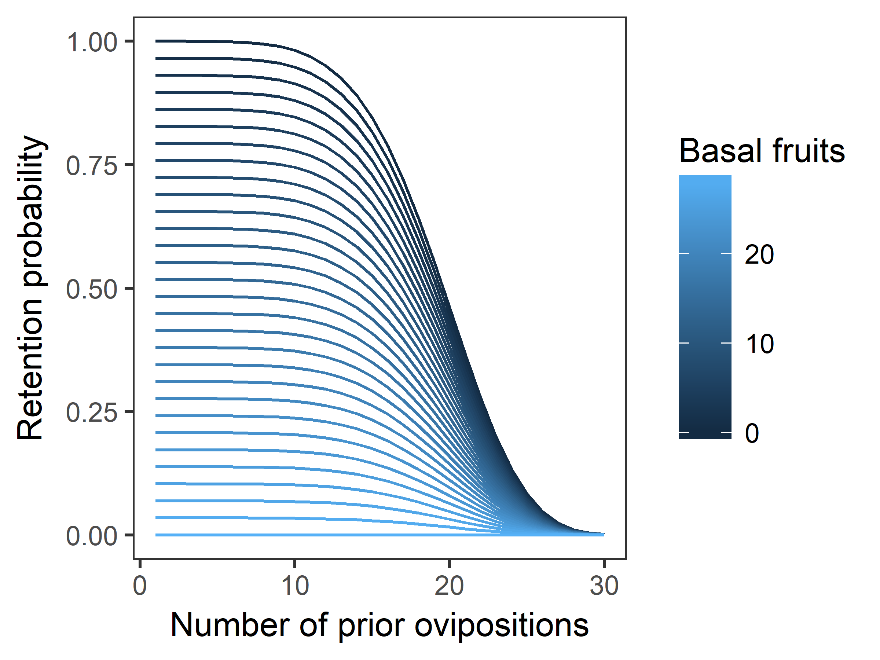


**Figure S2.2.** Probability of retaining flowers.

We simulated the following four scenarios: (1) *P_ovi_* and *λ* are independent of *B*, (2) *P_ovi_* decreases with *B*, but *λ* is independent of *B*, (3) *P_ovi_* is independent of *B*, but *λ* decreases with *B*, and (4) *P_ovi_* and *λ* decrease with *B*. For each scenario we simulated the fate of 10,000 inflorescences with 30 sequentially opening flowers each. Each simulation started with the bottom-most flower opening on all inflorescences. In our model, one flower per inflorescence opened each time step, and each flower was receptive for only one time step. Hence, flower position and time in the flowering season were correlated in our model, which is in line with the general flowering pattern of *Yucca* spp. Each time step the model cycled through every newly opened flower of all inflorescences and recorded the number of ovipositions per retained flower. For each flower the model went through the following sequence of events:

The model determined the number of moths arriving, *M*, by drawing a random number from a Poison distribution with a mean of 5. When more than one moth arrived (*M*>1), we assumed moths arrived one after another during the time step and not simultaneously. For each moth, we first determined whether the moth laid at least one egg by drawing a random number from a binomial distribution (yes/no) given by P_ovi_ (equation 1, or equation 2). In case of yes, we determined the number of ovipositions by drawing a random number from a zero-truncated Poisson distribution with the mean of 6 (constant) or given by equation 3. Once the model cycled through all arriving moths it determined whether a flower is retained. If a flower did not receive any ovipositions it was aborted because of lack of pollination (yucca moths are obligate pollinators of *Yucca* spp). If a flower received at least one oviposition, the model determined whether the flower was retained by drawing a random number from a binomial distribution (yes/no) given by *P_ret_* (equation 4).

We analyzed the simulation results by quantifying the number of eggs in fruits of top third flowers and the associated number of basal fruits. Assuming that the survival of eggs in retained flowers is not influenced by the number of basal fruits our simulation results show that only when moths decrease the number of ovipositions with increasing number of basal fruits, $\lambda(B)$ (equation 3) can we expect a negative relationship between number of emerging larvae and number of basal fruits (Figure S2.3). This supports our notion that the number of emerging larvae is a proxy for the number of ovipositions to test whether *T. yuccasella* will decrease the number of ovipositions in response to number of basal fruits.


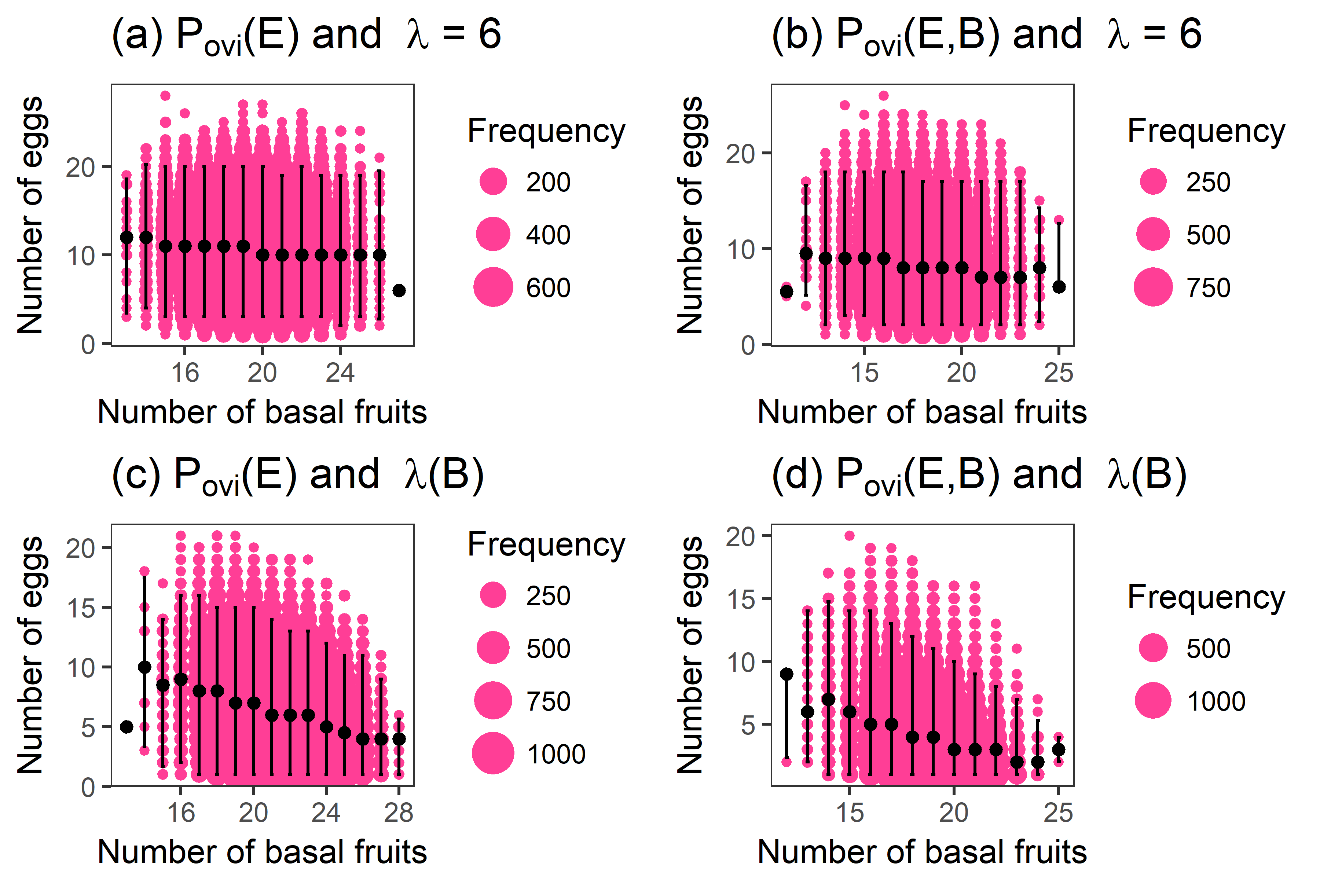


**Figure S2.3.** Predicted relationships between number of basal fruits and number of egg from simulations of four scenarios: (1) *P_ovi_* and *λ* are independent of *B*, (2) *P_ovi_* decreases with *B*, but *λ* is independent of *B*, (3) *P_ovi_* is independent of *B*, but *λ* decreases with *B*, and (4) *P_ovi_* and *λ* decrease with *B.* Points are fruits from simulated flowers, and point size is proportional to the frequency of points. Black points and error bars are median, and upper and lower 95% quantiles of number of larvae for each number of basal fruits, respectively.

References:

Humphries, S. & Addicott, J., 2000. Regulation of the mutualism between yuccas and yucca moths: intrinsic and extrinsic factors affecting flower retention. *Oikos*, 89(2), pp.329–339.

Huth, C.J. & Pellmyr, O., 1999. Yucca moth oviposition and pollination behavior is affected by past flower visitors: evidence for a host-marking pheromone. *Oecologia*, 119(4), pp.593–599.

Louda, S.M. et al., 2011. Priority resource access mediates competitive intensity between an invasive weevil and native floral herbivores. *Biological Invasions*, 13(10), pp.2233–2248.

Pellmyr, O. & Huth, C.J., 1994. Evolutionary stability of mutualism between yuccas and yucca moths. *Nature*, 372, pp.257–260.

Shapiro, J. & Addicott, J.F., 2004. Re-evaluating the role of selective abscission in moth/yucca mutualisms. *Oikos*, 105(3), pp.449–460.

**Appendix S3:**


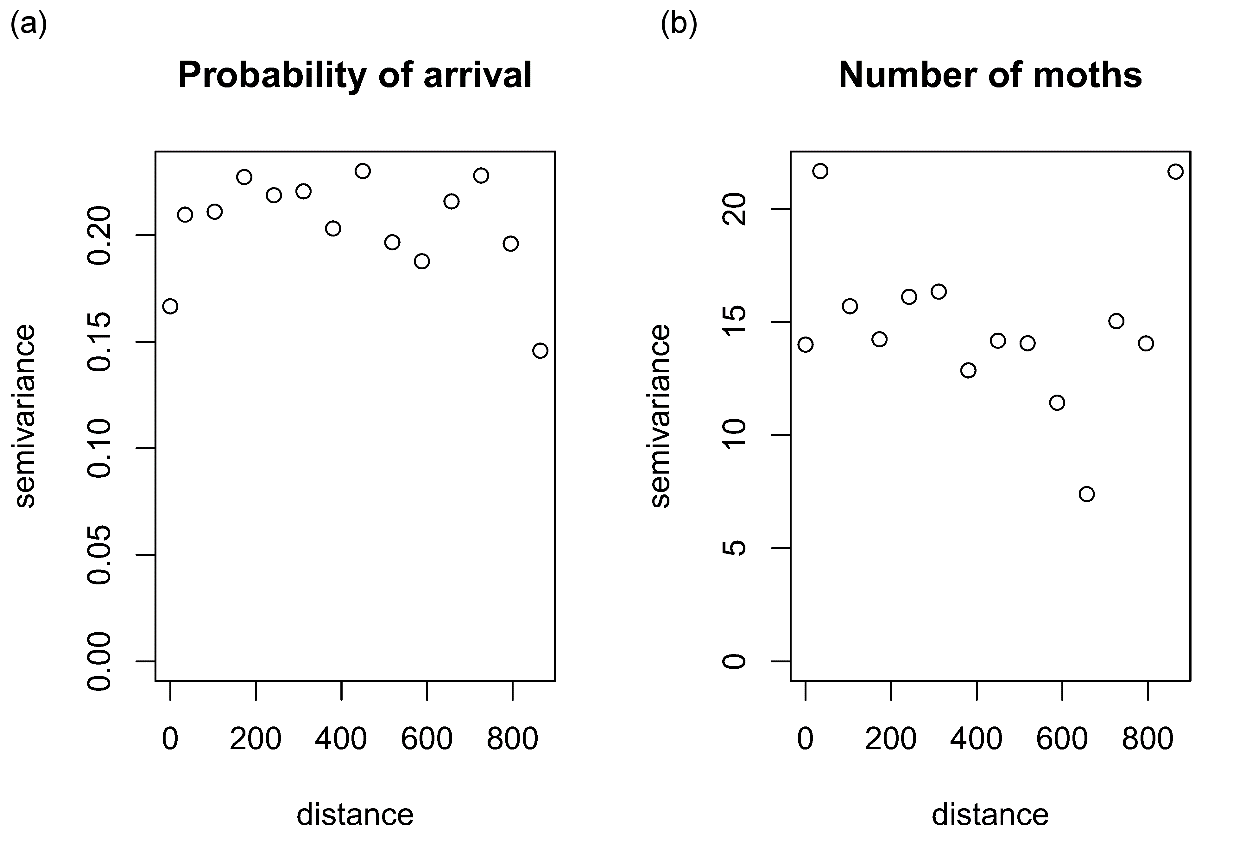


**Figure S3.1.** Semivariograms for the (a) probability and (b) number of *T. yuccasella* arriving at inflorescences at onset of flowering. A high nugget effect (semivariance at 0 distance on x-axis) compared to the sill (upper limit of the semivariogram) suggests a very little spatial autocorrelation in the probability and number of moths arriving at inflorescences at onset of flowering.

| **Table S1.** Distribution of number of trials carried out during the experiment. We discarded all the trials where moths did not exit their vials. | | | | | | |
| --- | --- | --- | --- | --- | --- | --- |
| Trial order | Inflorescence treatment | | | | | Total |
|  | no fruits | |  | basal fruits | |  |
|  | Analyzed | Discarded |  | Analyzed | Discarded |  |
| first | 9 | 0 |  | 9 | 3 | 21 |
| second | 6 | 0 |  | 5 | 2 | 13 |

| **Table S2.** Full model for the proportion of flowers with at least one oviposition during a trial with presence of basal fruits and trial order as predictors. The model is a generalized linear mixed-effects model with binomial error distribution, and with moth identity and night of trial as random effects. SE indicates standard errors. Variance of random effects - identity of moths and trial night is 0.50 ± 0.71 SD and 0.39 ± 0.62 SD, respectively. (n = 29 trials, from 18 moths over 11 trial nights). | | | | |
| --- | --- | --- | --- | --- |
| **Parameter** | **Estimated mean** | **SE** | **z value** | **Pr(>\|z\|)** |
| (Intercept) no basal fruits, first trials | -0.31 | 0.56 | -0.55 | 0.58 |
| basal fruits | -1.25 | 0.63 | -1.98 | 0.048 |
| second trials | -0.37 | 0.66 | -0.56 | 0.58 |

| **Table S3.** Full model for log-transformed total number of ovipositions in a trial with at least one oviposition, with presence of basal fruits and trial order as predictors. The model is a linear mixed-effects model with moth identity and night of trial as random effects. SE indicates standard errors. Standard deviation for random effects - trial night is 0.15, and identity of moths is 0.0001 with 0.9 residuals. (n = 16 trials from 11 moths over 10 trial nights). | | | | | |
| --- | --- | --- | --- | --- | --- |
| **Parameter** | **Estimated mean** | **SE** | **df** | **t value** | **Pr(>\|t\|)** |
| (Intercept) no basal fruits, first trials | 2.62 | 0.39 | 9 | 6.76 | 0.001 |
| basal fruits | -0.32 | 0.53 | 1 | -0.61 | 0.65 |
| second trials | -0.26 | 0.53 | 1 | -0.49 | 0.71 |

| **Table S4.** Final model for the proportion of flowers with at least one oviposition during a trial with presence of basal fruits as a predictor. The model is a generalized linear mixed-effects model with binomial error distribution, and with moth identity and night of trial as random effects. SE indicates standard errors. Variance of random effects - identity of moths and trial night is 0.27 ± 0.52 SD and 0.48 ± 0.7 SD, respectively. (n = 29 trials, from 18 moths over 11 trial nights). | | | | |
| --- | --- | --- | --- | --- |
| **Parameter** | **Estimated mean** | **SE** | **z value** | **Pr(>\|z\|)** |
| (Intercept) no basal fruits | -0.38 | 0.52 | -0.72 | 0.47 |
| basal fruits | -1.21 | 0.61 | -1.98 | 0.048 |

| **Table S5.** Model for log-transformed total number of ovipositions in a trial with at least one oviposition, with presence of basal fruits as a predictor. The model is a linear mixed-effects model with moth identity and night of trial as random effects. The predictor variable, trial order, was removed during backward selection. Standard deviation for random effects - trial night is 0.21, and identity of moths is 0.00005 with 0.91 residuals. (n = 16 trials from 11 moths over 10 trial nights). The model shows a non-significant effect of the presence of basal fruits on the number of ovipositions in trials where eggs were laid. Further model simplification during backward selection shows that this model is not significantly different from the null model (LRT = 0.39, p = 0.53). | | | | | |
| --- | --- | --- | --- | --- | --- |
| **Parameter** | **Estimated mean** | **SE** | **df** | **t value** | **Pr(>\|t\|)** |
| (Intercept) no basal fruits | 2.52 | 0.32 | 9 | 7.93 | <0.0001 |
| basal fruits | -0.30 | 0.51 | 2 | -0.59 | 0.61 |

| **Table S6.** Model for number of larvae emerging from fruits from top third flowers on naturally-pollinated inflorescences. The model is a generalized linear mixed-effects model with Poisson error distribution, and with inflorescence identity as a random effect. Predictor variables are number of basal fruits and year. Year was treated as a categorical variable. SE indicates standard errors. Variance of random effect - inflorescence identity is 0.33. (n = 243 fruits from 82 inflorescences). | | | | |
| --- | --- | --- | --- | --- |
| **Parameter** | **Estimated mean** | **SE** | **t value** | **Pr(>\|t\|)** |
| (Intercept) year 2014 | -0.2 | 0.49 | -0.4 | 0.69 |
| number of basal fruits | 0.01 | 0.04 | 0.33 | 0.74 |
| year 2015 | -1.87 | 0.52 | -3.60 | 0.0003 |
| year 2016 | -1.43 | 0.47 | -3.08 | 0.002 |

| **Table S7.** Full model for the probability of arrival of moths at onset of flowering with day of onset of flowering (onset day), number of flowers open at onset of flowers (flowers), basal diameter of the rosette (basal dia.), and the distance to the nearest tree (tree dist.) as predictors. The model is a generalized linear model with Binomial error distribution. (n = 111 inflorescences). | | | | |
| --- | --- | --- | --- | --- |
| **Parameter** | **Estimated mean** | **SE** | **z value** | **Pr(>\|z\|)** |
| (Intercept) | 3.28 | 1.85 | 1.77 | 0.08 |
| tree dist. | 0.005 | 0.03 | 0.15 | 0.88 |
| basal dia. | 0.02 | 0.02 | 0.97 | 0.33 |
| onset day | -0.33 | 0.09 | -3.61 | 0.0003 |
| flowers | 0.14 | 0.05 | 2.78 | 0.005 |

| **Table S8**. Candidate set of models for the probability of arrival of moths on the day of onset of flowering of an inflorescence (model syntax) with number of parameters (k), corrected Akaike Information criteria (AICc), differences in AICc (ΔAICc), Akaike weights (AICcWt), cumulative Akaike weights (Cum.Wt), and Log-likelihood (LL). GLM with binomial family of errors was used. Best approximating model is highlighted in grey. | | | | | | |
| --- | --- | --- | --- | --- | --- | --- |
| **Model syntax** | **k** | **AICc** | **ΔAICc** | **AICcWt** | **Cum.Wt** | **LL** |
| onset day + flowers | 3 | 118.38 | 0 | 0.57 | 0.57 | -56.08 |
| basal dia. + onset day + flowers | 4 | 119.59 | 1.21 | 0.31 | 0.88 | -55.61 |
| tree dist. + basal dia. + onset day + flowers | 5 | 121.76 | 3.38 | 0.10 | 0.98 | -55.6 |
| basal dia. + onset day | 3 | 126.6 | 8.22 | 0.01 | 0.99 | -60.19 |
| onset day | 2 | 127.32 | 8.94 | 0.01 | 1 | -61.61 |
| basal dia. + flowers | 3 | 134.26 | 15.88 | 0 | 1 | -64.02 |
| flowers | 2 | 135.03 | 16.64 | 0 | 1 | -65.46 |
| basal dia. | 2 | 136.52 | 18.14 | 0 | 1 | -66.21 |
| Null | 1 | 138.81 | 20.43 | 0 | 1 | -68.39 |
| tree dist. | 2 | 140.86 | 22.48 | 0 | 1 | -68.38 |

| **Table S9.** Final model for the probability of moths arriving at onset of flowering with day of onset of flowering (onset day) and number of flowers open at onset of flowers (flowers) as predictors. The model is a generalized linear model with binomial error distribution. (n = 111 inflorescences). | | | | |
| --- | --- | --- | --- | --- |
| **Parameter** | **Estimated mean** | **SE** | **z value** | **Pr(>\|z\|)** |
| (Intercept) | 4.61 | 1.29 | 3.59 | 0.0003 |
| onset day | -0.34 | 0.09 | -3.78 | 0.0002 |
| flowers | 0.15 | 0.05 | 3.01 | 0.003 |

| **Table S10.** Full model for the number of moths arriving at onset of flowering with smoothed day of onset of flowering (onset day), number of flowers open at onset of flowers (flowers), basal diameter of the rosette (basal dia.), and the distance to the nearest tree (tree dist.). The model is a generalized additive model with Poisson error distribution. (n = 76 inflorescences where moths arrived at onset of flowering). | | | | |
| --- | --- | --- | --- | --- |
| **Parameter** | **Estimated mean** | **SE** | **z value** | **Pr(>\|z\|)** |
| (Intercept) | -0.91 | 0.33 | -0.28 | 0.006 |
| tree dist. | -0.008 | 0.009 | -0.84 | 0.40 |
| basal dia. | -0.0006 | 0.004 | -0.14 | 0.89 |
| flowers | 0.04 | 0.01 | 3.10 | 0.002 |
| **Smoothing spline** |  |  | **Χ^2^** | **p-value** |
| s(onset day) |  |  | 31.08 | 0.0001 |

| **Table S11.** Candidate set of models for the number of moths arriving at inflorescences on day of onset of flowering (model syntax) with corrected Akaike Information Criteria (AICc), differences in AICc (ΔAICc), Akaike weights (AICcWt), cumulative Akaike weights (Cum.Wt), and Log-likelihood (LL). Generalized additive model with Poisson family of error was used with smoothing spline on day of onset of flowering. Best approximating model is highlighted in grey. | | | | | |
| --- | --- | --- | --- | --- | --- |
| **Model syntax** | **AICc** | **ΔAICc** | **AICcWt** | **Cum.Wt** | **LL** |
| s(onset day) + flowers | 396.51 | 0 | 0.69 | 0.69 | -188.54 |
| basal dia. + s(onset day) + flowers | 399.02 | 2.51 | 0.20 | 0.89 | -188.53 |
| tree dist. + basal dia. + s(onset day) + flowers | 401.06 | 4.55 | 0.07 | 0.96 | -191.85 |
| s(onset day) | 402.94 | 6.43 | 0.03 | 0.99 | -208.22 |
| basal dia. + s(onset day) | 405.19 | 8.68 | 0.01 | 1 | -216.07 |
| flowers | 420.60 | 24.09 | 0 | 1 | -215.37 |
| basal dia. + flowers | 422.23 | 25.72 | 0 | 1 | -188.54 |
| Null | 434.19 | 37.68 | 0 | 1 | -188.21 |
| basal dia. | 434.90 | 38.39 | 0 | 1 | -191.73 |
| tree dist. | 435.07 | 38.56 | 0 | 1 | -208.22 |

| **Table S12.** Final model for the number of moths arriving at onset of flowering with smoothed day of onset of flowering (onset day) and number of flowers open at onset of flowers (flowers) as predictors. The model is a generalized additive model with Poisson error distribution. (n = 76 inflorescences where moths arrived at onset of flowering). | | | | |
| --- | --- | --- | --- | --- |
| **Parameter** | **Estimated mean** | **SE** | **z value** | **Pr(>\|z\|)** |
| (Intercept) | 0.8 | 0.14 | 5.74 | <0.0001 |
| flowers | 0.04 | 0.01 | 3.27 | 0.001 |
| **Smoothing spline** | |  | **Χ^2^** | **p-value** |
| s(onset day) |  |  | 32.02 | <0.0001 |
